# *p53* mutation, deprivation and poor prognosis in primary breast cancer

**DOI:** 10.1038/sj.bjc.6605540

**Published:** 2010-01-26

**Authors:** L Baker, P R Quinlan, N Patten, A Ashfield, L-J Birse-Stewart-Bell, C McCowan, J-C Bourdon, C A Purdie, L B Jordan, J A Dewar, L Wu, A M Thompson

**Affiliations:** 1 Department of Surgery and Molecular Oncology, Dundee University, Ninewells Hospital and Medical School, Dundee DD1 9SY, UK; 2 Roche Molecular Systems, 4300 Hacienda Drive, Pleasanton, CA 94588, USA; 3 Health Informatics Centre, Dundee University, Mackenzie Building, Ninewells Hospital and Medical School, Dundee DD2 4BF, UK; 4 Department of Pathology, Ninewells Hospital and Medical School, Dundee DD1 9SY, UK; 5 Department of Oncology, Ninewells Hospital and Medical School, Dundee DD1 9SY, UK

**Keywords:** *p53*, primary breast cancer, socio-economic deprivation, overall survival, disease-free survival

## Abstract

**Background:**

The deprivation gap for breast cancer survival remains unexplained by stage at presentation, treatment, or co-morbidities. We hypothesised that *p53* mutation might contribute to the impaired outcome observed in patients from deprived communities.

**Methods:**

*p53* mutation status was determined using the Roche Amplichip research test in 246 women with primary breast cancer attending a single cancer centre and related to deprivation, pathology, overall, and disease-free survival.

**Results:**

*p53* mutation, identified in 64/246 (26%) of cancers, was most common in 10 out of 17 (58.8%) of the lowest (10th) deprivation decile. Those patients with *p53* mutation in the 10th decile had a significantly worse disease-free survival of only 20% at 5 years (Kaplan–Meier logrank *χ*^2^=6.050, *P*=0.014) and worse overall survival of 24% at 5 years (Kaplan–Meier logrank *χ*^2^=6.791, *P*=0.009) than women of deciles 1–9 with *p53* mutation (*c.f.* 56% and 72%, respectively) or patients in the 10th decile with wild-type *p53* (no disease relapse or deaths).

**Conclusion:**

*p53* mutation in breast cancer is associated with socio-economic deprivation and may provide a molecular basis, with therapeutic implications, for the poorer outcome in women from deprived communities.

Deprivation has been linked to a wide range of disease (Banks *et al*, 2006) and to poor outcome for patients diagnosed with cancer, including breast cancer (Schrijvers *et al*, 1995; Twelves *et al*, 2001; Thomson *et al*, 2004; Mullee *et al*, 2004), colorectal cancer (McMillan *et al*, 2003), and melanoma (MacKie and Hole, 1996).

Although the incidence of breast cancer has risen more rapidly in women with a high socio-economic status than those from deprived areas, the difference in survival – the ‘deprivation gap’ – has not changed (Quinn *et al*, 2008) and cannot be attributed to the higher uptake of breast screening in higher socio-economic groups (Thomson *et al*, 2004). Possible explanations include:

- later presentation (Kogevinas and Porta, 1997), although not substantiated in all populations (Sant *et al*, 1998);
- under-treatment, evident in Scotland in the 1980s (Twelves *et al*, 2001) but not pertinent by 1994 (Thomson *et al*, 2004);
- reproductive factors, such as age at first pregnancy, menopause, and use of hormone replacement therapy, which even where highlighted would not account for all the difference (Thomson *et al*, 2001);
- biological differences between breast cancer arising in women according to socio-economic group. For example, a higher incidence of oestrogen receptor (ER)-positive tumours in higher social groups in some (Thomson *et al*, 2001) but not all series (Al Murri *et al*, 2004).

Given the uncertainty for the underlying basis for the deprivation gap, we considered the *p53* gene a candidate molecular marker that might account for the biological differences.

Briefly, *p53* has pleiotropic functions including responses to cellular stresses leading to cell cycle arrest, apoptosis or cellular senescence (Staples *et al*, 2008). *p53* also fulfills functions during development in normal tissues (Vousden and Lane, 2007) and in response to inflammation (Staib *et al*, 2005). The role of *p53* in breast cancer has been the subject of much debate (Staples *et al*, 2008) with recent clarity that *p53* mutation is a marker of biologically aggressive disease (Olivier *et al*, 2006; Staples *et al*, 2008) and response to therapy (Staples *et al*, 2008).

Given the uncertainties around the reasons for the poorer outcome for women with breast cancer from deprived areas and the continuing interest around the roles of *p53* in breast cancer, we set out to test the hypothesis that breast cancer in deprived women is biologically more aggressive than breast cancer in less deprived women and that *p53* mutation may account, at least in part, for this association.

## Materials and methods

### Patients and tissues

A total of 246 Caucasian patients undergoing resection for primary, previously untreated, operable breast cancer tissue between 1997 and 2001, with frozen tissue stored in the regional tissue bank and a minimum follow-up period of at least 5 years (or to death), but otherwise unselected, were studied. All women, regardless of social category, were diagnosed and treated at the regional cancer centre served by a single multi-disciplinary team. The total sample size of 246 was estimated on the basis of deprivation deciles of minimum size 15 patients. Local research ethics tissue bank committee approval of the project was obtained.

Clinical and pathological data were collected prospectively and included pathological tumour size, histological grade, ER, progesterone receptor and HER2 (Human Epidermal growth factor Receptor 2) receptor status, pathological lymph node status, adjuvant therapies, recurrence(s) and survival.

Breast tissue was macro-dissected by a specialist breast pathologist immediately after operation and tumour was snap-frozen in liquid nitrogen before storage at −80°C. Total genomic DNA was isolated using MagAttract DNA Mini M48 Kit on a BioRobotM48 (Qiagen, Crawley, West Sussex, UK) according to the manufacturer's protocol. The quantity and purity of DNA was determined using a NanoDrop ND-1000 spectrophotometer (Labtech International, Ringmer, East Sussex, UK).

### AmpliChip *p53* Test

*p53* mutation status was determined using the Roche *p53* Amplichip research test (Roche Molecular Systems, Pleasanton, CA, USA) from 100 ng of purified genomic DNA extracted from homogenised fresh frozen tumour tissues. The AmpliChip *p53* microarray consists of over 33 000 probe sets of more than 220 000 individual oligonucleotides tiled for a total of 1268 nucleotide positions of coding regions of exons 2–11. A single probe set for an interrogating base position includes five probes: one probe to hybridise to the WT, three probes to detect three possible single base-pair mutations, and one probe to detect single deletion. There are at least 24 probe sets for each nucleotide position, including both sense and anti-sense probe sequences. The *p53* mutation status was determined by a *p53* Mutation Detection Algorithm developed for research use by Roche Molecular Systems, which is designed to detect single base-pair substitutions and single base-pair deletions of a sample in a background of WT *p53* DNA probe intensities.

### Deprivation data

Deprivation was calculated using the Carstairs index of socio-economic status (Carstairs and Morris, 1991) for patients who were diagnosed with breast cancer between 1997 and 2001. The Carstairs index produces deprivation scores for postcode sectors using the standard UK postcode minus the last two characters, so large areas are grouped together under a single deprivation score. The Information Services Division, Scotland (Bishop *et al*, 2004) has subdivided each of these areas to produce a decile score for each full postcode in Scotland ranging from 1 (most affluent) to 10 (most deprived). This decile score allows the precise linkage of patients to the correct deprivation score, and was adopted for this study.

### Data analyses

Initial analyses indicated that the deprivation extremes were more likely to yield statistically significant data; indeed, deprivation categories 2–9 rarely produced statistically differing results. Moreover, deprivation category 1 did not produce significant results compared with categories 2–9, and the deprivation results were, therefore, subsequently grouped as 1–9 and 10, unless otherwise stated.

Data were analysed for three cohorts of patients: (1) all patients in the study, (2) all patients with a *p53* mutation (*p53*m), and (3) all patients that did not have a *p53* mutation (WT; *p53*WT).

Additional analyses were performed according to histological tumour grade (Bloom and Richardson, 1957) (graded by a specialist consultant breast pathologist); pathological tumour size (pT1, tumours <2 cm *vs* pT2 and pT3 cancers – tumours ⩾2 cm); ER status (as ER negative: 0–3 *vs* ER positive: 4–18 by the Quickscore method (Detre *et al*, 1995)); HER2 positive (HER2 2+ and gene amplified on fluorescence *in situ* hybridisation or HER2 3+ on immunohistochemistry); and triple negative (as ER, progesterone receptor and HER2 negative) or not triple negative.

Treatments were analysed to assess whether survival and recurrence intervals were influenced by differences in treatment. Recurrence was defined as clinical, radiological or pathological evidence of disease recurrence. Survival was calculated from the date of surgery to the date of death (last recorded hospital visit for censored data). All deaths that were not attributable to breast cancer were censored at the date of death. Accordingly, the primary end points were breast cancer-specific overall survival and breast cancer-specific disease-free survival (respectively abbreviated to OS and DFS throughout).

Tests for statistical significance between ordinal variables were performed by two-sided Fisher's exact tests (2FET) and non-parametric Kaplan–Meier survival analyses (KM) using Mosaic, an internally developed statistical analysis system (implemented in Matlab, Mathworks, Natick, MA, USA; Version 6.5 Release 13). For all analyses, the null hypothesis was rejected at an *α*-level of 10% (*P*>0.10), observations were considered to be marginal (i.e. worthy of further analysis) for an *α*-level between 5 and 10% (0.05⩽*P*<0.10), and significant at a 5% *α*-level (*P*<0.05). Further analyses were conducted using binary logistic regression (BLR) with associated odds ratios (ORs) using Minitab (Minitab State College, PA, USA, Release 14.1) and Cox's proportional hazards regression model with associated risk ratios (RRs).

## Results

Deprivation category, clinical and pathological data, and *p53* mutation status were successfully ascertained in all 246 patients (Table 1). The observed frequency of most variables was close to the expected in each decile (as calculated by their *χ*^2^ scores), and there was no gradation across deprivation deciles for any variable. However, several variables in deprivation category 10 registered significantly high or low values.

Overall, 109 of the 234 patients with tumour grade assessment were grade 3 (46.6%) with significantly more (12 out of 17; 70.6%) grade 3 tumours in the lowest deprivation decile (*P*=0.046, 2FET; OR=3.0(1.0, 8.7); power=0.54). For HER2 status, 33 out of 230 tested were HER2 positive (14.3%), with more HER2-positive patients in deprivation category 10 (6/16; 37.5%) than expected (*P*=0.015, 2FET; OR=4.2(1.4, 12.4); power=0.72). HER2 positivity was also significantly associated with *p53* mutation (*P*=0.003, 2FET; OR=3.3(1.5,7.0); power=0.85). Age, tumour size, nodal status, ER, progesterone receptor and triple-negative cancers were not significantly associated with deprivation category.

### *p53* mutation

*p53* mutation was detected in 64 cancers from 246 patients (26.0%), excluding the *p53* polymorphism at amino-acid 72 as a neutral change in terms of *p53* function. *p53* mutation was significantly associated with a decrease in OS (34% *vs* 10% dead – *P*<0.0001, 2FET; OR=0.2(0.1,0.5); power=0.98) and DFS (47% *vs* 18% relapsed – *P*<0.0001, 2FET; OR=0.2(0.1,0.5); power=0.99). Tumour *p53* mutation was significantly associated with grade 3 cancer (*P*<0.0001, 2FET; OR=10.3(4.9,21.8); power=1.00); axillary lymph node metastasis (*P*=0.004, 2FET; OR=2.4(1.3,4.3); power=0.84); ER-negative tumours (*P*<0.0001, 2FET; OR=4.9(2.6,9.0); power=1.00); HER2 expression (*P*=0.003, 2FET; OR=3.3(1.5,7.0); power=0.85) and triple-negative tumours (*P*=0.001, 2FET; OR=3.3(1.7,6.5); power=0.92). There was no association between *p53* mutation status and pathological tumour size or patient age.

### Univariate analyses

Both OS in deprivation category 10 (*P*=0.012, 2FET; OR=0.2(0.1,0.7); power=0.74); and DFS (*P*=0.042, 2FET; OR=0.3(0.1,0.9); power=0.57) were worse than expected. Patients in deciles 1–9, but not decile 10, were significantly more likely to have a relapse and die if they had a grade 3 tumour, large cancer, ER-negative, HER2-positive or triple-negative cancer (KM: logrank *χ*^2^⩾5.650, *P*⩽0.017 in each case, Table 2). Patients with axillary metastases were more likely to have a relapse or die from breast cancer irrespective of deprivation category, with the notable exception that patients in deprivation category 10 were significantly more likely to have a relapse, but were not significantly more likely to die (*χ*^2^⩾4.620, *P*⩽0.032 in each case – excepted *χ*^2^=3.080, *P*=0.079, Table 2). There was no association between any of the treatment regimes and deprivation.

Tumour *p53* mutation was more common in deprivation decile 10 (10 out of 17 patients, 58.8%) than WT *p53* (*P*=0.003, 2FET; OR=4.6(1.7,12.7); power=0.87). In the patients with *p53* mutation, 27.8% (15 out of 54) of the patients in deprivation group 1–9 died, whereas 70.0% (7 out of 10) of those in decile 10 died (*P*=0.025, 2FET; OR=6.1(1.4,26.6); power=0.74). Similarly, among *p53* mutant cancers 40.7% (22 out of 54) of patients in deprivation groups 1–9 relapsed compared with 80% (8 out of 10) of those in the worst deprivation category (*P*=0.025, 2FET; OR=6.1(1.4,26.6); power=0.74; and *P*=0.036, 2FET; OR=5.8(1.1,30.0); power=0.65; respectively). However, in the *p53* WT cohort, there was no such association between deprivation and OS or DFS; indeed, all seven patients with *p53* WT cancers in deprivation category 10 survived and remained disease free at a median follow-up of 7.2 years (95% CI: 5.5–8.1 years).

### Multivariate analyses

The analyses of BLRs and Cox's logistic regressions (Table 3) were also performed. Death and recurrence were chosen as the response variables so that most of the OR and RR values (BLR and Cox's proportional hazards regression, respectively) would be greater than unity (OS and DFS OR and RR values and 95% CI values may be obtained by inversion). Results of BLRs and Cox's proportional hazards regressions were in agreement with all analyses, and as such, the results of BLR analyses have been excluded from Table 3.

In the first two sets of analyses (top half of Table 3), deprivation groups 1–9 and 10 were input as binary variables, as were *p53*m/WT for the prediction of death and recurrence (left and right of Table 3, respectively). In the second set of analyses (bottom half of Table 3), the influence on death and recurrence of deprivation category 10 patients who had *p53*-mutated cancer was explored. Deprivation category 10 was a strong predictor of death and recurrence, as were large tumours, ER negativity and nodal status (*P*⩽0.041 in each case). Interestingly, *p53* mutation was strongest as a predictor of disease recurrence, but less strong as a predictor for death.

When patients were grouped according to deprivation category 10 with a *p53* mutation (*vs* all other patients), the ability to predict death or recurrence became very strong and highly significant (*β*⩾±2.18, *P*⩽0.0001 in all cases). The OR and RR values for death and recurrence were large (OR=33.4(5.2,215.0) and OR=19.6(3.4,114.2) and RR=12.4(4.8,32.3) and RR=8.8(3.6,21.7), respectively). Tumour size, ER status and nodal status all retained large *β* coefficients and remained significant predictors of death and recurrence (*β*⩾±0.90, *P*⩽0.026 in all cases).

In addition, although there was no association between any of the treatment regimes and deprivation in univariate analyses, all multivariate analyses were re-run with various treatment regimes included as binary variables (data not shown). Treatment regimes were not significant, and were therefore not associated with the outcome variables.

### Kaplan–Meier survival analyses

Patients from the worst deprivation decile were significantly more likely to have a relapse or die than those in deprivation groups 1–9 (5-year OS: 57% *vs* 86% KM: logrank *χ*^2^=7.827, *P*=0.005 for OS and KM: logrank *χ*^2^=4.450, *P*=0.035 for DFS). Similarly, patients with a mutant *p53* were more likely to relapse or die compared with those with WT *p53* (5-year OS: 65% *vs* 90% KM: logrank *χ*^2^=21.267, *P*<0.0001 for OS and KM: logrank *χ*^2^=23.308, *P*<0.0001 for DFS).

**Figure 1:**
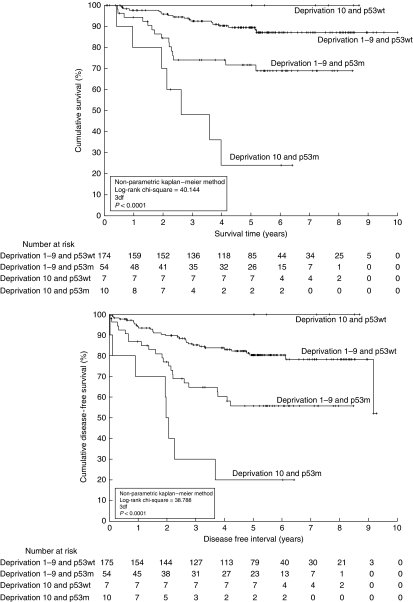
Non-parametric Kaplan–Meier plots for overall survival (top) and disease-free survival (bottom) for four patient groupings – those in deprivation category 10 *vs* decile 1–9, and those having a *p53* mutation *vs* wild-type *p53*. The logrank *χ*^2^ statistic (with associated degrees of freedom and *P*-value) was used to assess the statistical significance of difference in survival and disease-free chances. Patients with a *p53* mutation had 5-year survivals of 72 and 24% (deprivation groups 1–9 and 10, respectively) and corresponding 5-year disease-free survivals of 56 and 20%. Patients with wild-type *p53* had 89 and 100% chance of surviving until 5 years (deprivation groups 1–9 and 10, respectively), with corresponding 5-year disease-free survivals of 80 and 100%.

Patients were divided into four groupings – each of deprivation groups 1–9 and 10 with each of *p53* mutation and WT – and plotted as KM analyses in Figure 1 (OS and DFS, top and bottom, respectively).

Within the decile groups (1–9 and 10), OS and DFS were both – as expected – better in the *p53* WT patients compared with the mutant (decile 1–9: 5-year OS: 89% *vs* 72% decile 10: 5-year OS: 100% *vs* 24% decile 1–9 KM: logrank *χ*^2^=9.958, *P*=0.002 and KM: logrank *χ*^2^=12.098, *P*=0.0005 for OS and DFS, respectively; decile 10 KM: logrank *χ*^2^=8.011, *P*=0.005 and KM: logrank *χ*^2^=9.177, *P*=0.002, OS and DFS, respectively).

Within the *p53*-mutant patients, those in deprivation decile 10 were more likely to have a relapse or die compared with those in deciles 1–9 (*p53*m: 5-year OS: 24% *vs* 72% *p53*m 5-year DFS: 20% *vs* 55% KM: logrank *χ*^2^=6.791, *P*=0.009 and KM: logrank *χ*^2^=6.050, *P*=0.014 for OS and DFS, respectively). In contrast, within the WT *p53* patients, there was no statistically significant difference between decile group 1–9 and 10 for either OS or DFS (KM: logrank *χ*^2^=0.917, *P*=0.338 and KM: logrank *χ*^2^=1.626, *P*=0.202 for OS and DFS, respectively).

## Discussion

This study demonstrates the new finding that a worse survival and shorter disease free interval in breast cancer for the most socio-economically deprived patients (deprivation decile 10) is associated with tumour *p53* mutation. This suggests the intriguing possibility of a causal link between the molecular basis of breast cancer exemplified by *p53* mutations and extreme deprivation, and that the biological features of breast cancer may contribute substantially to the perceived deprivation gap in survival compared with stage of presentation, co-morbidities or treatment differences (Kelsey *et al*, 1981; Schrijvers *et al*, 1997; Twelves *et al*, 2001; Taylor and Cheng, 2003; Thomson *et al*, 2004; Mullee *et al*, 2004).

*p53* mutation in this series of patients was associated, as anticipated, with high tumour grade, axillary node metastasis, ER-negative cancers and HER2-positive cancers (Staples *et al*, 2008). Although – again as expected – tumour *p53* mutation conferred an increased chance of relapse and death compared with having a WT *p53* tumour (Olivier *et al*, 2006; Staples *et al*, 2008), it seems that deprivation is associated with a worse prognosis even amongst those with *p53* mutation. Interestingly, for WT *p53* patients, this does not hold: all patients in deprivation category 10 with WT *p53* breast cancers remained disease free and survived. Reasons for this extreme difference are unclear; it is unlikely that patient selection (on the basis of the availability of tissue rather than consecutive patients by decile of residence) was responsible but suggests that the combination of *p53* mutation in breast cancer and extreme deprivation prejudices survival.

The influence of established prognostic features of breast cancer when comparing extreme deprivation (decile 10) patients with higher socio-economic status could not be excluded as contributors in the current series, with the biological features of poorer prognostic cancers (higher grade, ER negative and HER2 positive) in general more evident in the decile 10 patients. Moreover, the relationship between deprivation and ER positivity (Thomson *et al*, 2001) was not, as others have also found (Al Murri *et al*, 2004), confirmed in this study. The effects of treatment were also considered. The response of the *p53* network in breast cancer to chemotherapies and radiation therapy may be dependent on the *p53* status of the cancer (Al Murri *et al*, 2004; Staples *et al*, 2008). The increased presence of *p53* mutations in the worst deprivation decile suggests such cancers may also be resistant to treatment and has implications for the types of chemotherapy that may be most effective (Staples *et al*, 2008).

From the results of multivariate analyses, patients from the worst deprivation group were significantly more likely to have a relapse and die from breast cancer, even after adjusting for other associated variables. Interestingly, *p53* mutation status in itself was significantly associated with death when using Fisher's exact tests and Kaplan–Meier analyses, but not when analysed by binary logistic regression or Cox's regression. This suggests that *p53* mutation status has a strong interaction with (at least) one other variable in the model (i.e. a dominant ‘partner’); therefore an interaction between *p53* mutation and deprivation decile 10 was sought. The *β*-coefficient of the interaction between *p53* mutation and deprivation category 10 was positive and statistically significant (*β*=2.52, *P*<0.0001 and *β*=2.18, *P*<0.0001 for death and recurrence, respectively). Moreover, the impact of the interaction was substantially greater than the additive effect (summation of the RR values) of the two variables alone, indicating a potentially synergistic relationship between deprivation and *p53* mutation both for survival and disease recurrence.

Among the pleiotropic roles of *p53*, *p53* has a pivotal node in the inflammatory stress response pathway (Staib *et al*, 2005). In addition post-translational modification of *p53* by reactive nitrogen species (Hofseth *et al*, 2003) may lead to the selective clonal expansion of mutated *p53* cells (Hofseth *et al*, 2003). As originally proposed by Virchow in the 19th century, links between inflammation and cancer have been established for both malignant and pre-malignant conditions (Hussain and Harris, 2007). However, although *p53* mutations have been identified in cancer-prone inflammatory diseases, including premalignant inflammatory conditions of the gastrointestinal tract (Hussain *et al*, 2000), to date no similar association has been proposed in breast cancer. Although the disparate range of *p53* mutations identified (data not shown) suggests a single agent is unlikely to be causative, patients in deprived communities do have a chronic inflammatory response that has been linked to breast cancer (Al Murri *et al*, 2004), colorectal cancer (McMillan *et al*, 2003) and increased coronary heart disease (Ridker *et al*, 2000; O’Reilly *et al*, 2006). Most recently, evidence that raised circulating markers of chronic inflammation (C-reactive proteins and serum amyloid A) have a threshold effect on breast cancer survival has emerged (Pierce *et al*, 2009). The tentative association explored between elevated C-reactive protein, serum amyloid A with ER- and progesterone receptor-negative cancers indirectly suggests further merit in pursuing a link between such inflammatory markers and *p53*. We recognise that our findings need to be confirmed in another population of breast cancer patients, ideally, one with data on inflammatory markers and the standard breast cancer data.

*p53* mutation, with potential aberrant inflammatory stress responses and therapeutic consequences, may account – at least in part – for the poor prognosis in women with breast cancer from the most deprived socio-economic background. This underlines the need to address the broader environment and social context of patient care along-side our increasing molecular understanding of cancer.

## Figures and Tables

**Table 1:** Study characteristics of the breast cancer clinical and pathological variables grouped by deprivation 1–9 and 10 and by *p53* mutation status

		**p53m**						**p53WT**					
		**Deprivation 1–9**			**Deprivation 10**			**Deprivation 1–9**			**Deprivation 10**		
	**Total**	***N***	**%**	***χ* ^2^**	***N***	**%**	***χ* ^2^**	***N***	**%**	***χ* ^2^**	***N***	**%**	***χ* ^2^**
Total number	246	54	—	—	10	—	—	175	—	—	7	—	—
Alive	205	39	72.2	−0.80	3	30.0	−**3.41**	156	89.1	+0.71	7	100.0	+0.23
Dead	41	15	27.8	**+4.00**	7	70.0	**+17.07**	19	10.9	−**3.54**	0	0.0	−**1.17**
Disease free	184	32	59.3	−**1.74**	2	20.0	−**4.01**	143	81.7	**+1.12**	7	100.0	+0.59
Recurred	62	22	40.7	**+5.17**	8	80.0	**+11.91**	32	18.3	−**3.32**	0	0.0	−**1.76**
													
*Tumour grade*													
1	34	0	0.0	−**7.41**	0	0.0	−**1.45**	31	18.7	**+1.96**	3	42.9	**+3.87**
2	91	9	17.6	−**5.92**	1	10.0	−**2.15**	80	48.2	**+3.69**	1	14.3	−**1.09**
3	109	42	82.4	**+14.01**	9	90.0	**+4.05**	55	33.1	−**6.45**	3	42.9	−0.02
													
Not known	12	—	—	**—**	—	—	—	—	—	—	—	—	—
													
*Tumour size*													
<2 cm	170	34	65.4	−0.18	8	80.0	+0.14	122	70.5	+0.00	6	85.7	+0.24
⩾2 cm	72	18	34.6	+0.41	2	20.0	−0.32	51	29.5	−0.00	1	14.3	−0.56
													
Not known	4	—	—	—	—	—	—	—	—	—	—	—	—
ER+	175	24	44.4	−**5.50**	5	50.0	−0.64	140	80.5	**+1.99**	6	85.7	+0.20
ER−	70	30	55.6	**+13.76**	5	50.0	**+1.61**	34	19.5	−**4.97**	1	14.3	−0.50
Not known	1	—	—	**—**	—	—	**—**	—	—	**—**	—	—	—
HER2+	33	14	28.6	**+6.91**	2	20.0	+0.22	13	7.9	**−4.81**	4	66.7	**+11.45**
HER2−	197	35	71.4	**−1.16**	8	80.0	−0.04	152	92.1	+0.81	2	33.3	**−1.92**
Not known	16	—	—	**—**	—	—	—	—	—	—	—	—	**—**
TrNeg	45	17	34.7	**+5.73**	4	40.0	**+2.13**	24	14.5	**−2.13**	0	0.0	**−1.17**
Not TrNeg	185	32	65.3	**−1.39**	6	60.0	−0.52	141	85.5	+0.52	6	100.0	+0.29
Not known	16	—	—	**—**	—	—	—	—	—	—	—	—	—
Node+	117	35	64.8	**+3.38**	6	60.0	+0.33	74	42.3	**−1.02**	2	28.6	−0.53
Node−	129	19	35.2	**−3.07**	4	40.0	−0.30	101	57.7	+0.93	5	71.4	+0.48
													
*Age (years)*													
<40	14	4	7.4	+0.28	0	0.0	−0.57	8	4.6	−0.39	2	28.6	**+6.44**
40–49	34	10	18.5	+0.86	2	20.0	+0.28	21	12.0	−0.42	1	14.3	+0.00
50–59	69	13	24.1	−0.30	3	30.0	+0.01	51	29.1	+0.07	2	28.6	+0.00
⩾60	129	27	50.0	−0.06	5	50.0	−0.01	95	54.3	+0.11	2	28.6	−0.76
													
Median age (years)	61.30	58.79 (28.72–88.48)			61.15 (43.58–84.25)			61.94 (33.53–88.99)			55.51 (39.69–87.78)		
Median FU (years)	4.96	4.94 (0.06–8.47)			2.44 (0.41–6.42)			4.96 (0.10–10.00)			7.20 (5.02–8.70)		
Median DF (years)	4.74	4.10 (0.02–8.47)			2.02 (0.02–6.42)			4.83 (<0.01–9.29)			7.20 (5.02–8.70)		

Abbreviations: DF=disease-free survival; ER=oestrogen receptor; FU=follow-up.

Percentages may not sum to 100 due to rounding. The *χ*^2^ score, calculated across all four *p53*/deprivation categories, indicates the deviation of the observed from the expected frequency, in which a high score signifies a large deviation and a *χ*^2^ score greater than unity is deemed ‘of interest’, and highlighted in bold type. The *χ*^2^ score is marked with a ‘+’ if the observed frequency exceeds the expected, and with a ‘−’ otherwise. Median ages and follow-up were not significantly different for any of the patient groupings (p⩾0.342 in all cases; two-sample *t*-test and Mann–Whitney *U*-test).

**Table 2:** Results of Kaplan–Meier overall survival and disease-free survival analyses for all patients, patients in the *p53* mutant and in the *p53* wild-type cohorts

		**Survival**						**Disease free**					
		**All patients**		**p53 m patients**		**p53WT patients**		**All patients**		**p53 m patients**		**p53WT patients**	
	**Dep. group**	***χ* ^2^**	***P***	***χ* ^2^**	***P***	***χ* ^2^**	***P***	***χ* ^2^**	***P***	***χ* ^2^**	***P***	***χ* ^2^**	***P***
*p53* mutation (*vs* WT)	All	**21.27**	**<0.0001**	—	—	—	—	**23.31**	**<0.0001**	—	—	—	—
	1–9	**9.96**	**0.002**	—	—	—	—	**12.10**	**0.001**	—	—	—	—
	10	**8.01**	**0.005**	—	—	—	—	**9.18**	**0.002**	—	—	—	—
Tumour grade (1–2 *vs* 3)	All	**22.33**	**<0.0001**	2.95^a^	0.086^a^	**9.23**	**0.002**	**17.38**	**<0.0001**	3.22^a^	0.073^a^	2.75	0.097
	1–9	**18.81**	**<0.0001**	3.55^a^	0.059^a^	**9.58**	**0.002**	**13.96**	**0.0002**	**3.94** a	**0.047** a	2.97	0.085
	10	1.51	0.219	0.01^a^	0.937^a^	N/A^b^	N/A^b^	1.31	0.252	**8.20** a	**0.004** a	N/A^b^	N/A^b^
Tumour size (<2 cm *vs* ⩾2 cm)	All	**10.13**	**0.001**	0.32	0.574	**14.95**	**0.0001**	**8.06**	**0.005**	2.20	0.138	**5.11**	**0.024**
	1–9	**11.01**	**0.001**	0.28	0.596	**14.32**	**0.0002**	**8.25**	**0.004**	2.30	0.130	**4.75**	**0.029**
	10	1.08	0.298	1.07	0.300	N/A^b^	N/A^b^	2.05	0.152	**9.54**	**0.002**	N/A^b^	N/A^b^
ER (+ *vs* −)	All	**31.65**	**<0.0001**	**5.75**	**0.017**	**13.79**	**0.0002**	**25.06**	**<0.0001**	**5.38**	**0.020**	**6.54**	**0.011**
	1–9	**27.83**	**<0.0001**	**5.81**	**0.016**	**13.66**	**0.0002**	**22.71**	**<0.0001**	**6.80**	**0.009**	**6.47**	**0.011**
	10	3.40	0.065	0.97	0.326	N/A^b^	N/A^b^	1.80	0.180	0.14	0.704	N/A^b^	N/A^b^
HER2 (+ *vs* −)	All	3.38	0.066	0.24	0.626	**4.62**	**0.032**	**6.24**	**0.013**	0.67	0.414	0.74	0.389
	1–9	**5.65**	**0.017**	0.15	0.702	**8.71**	**0.003**	**8.33**	**0.004**	0.59	0.441	2.61	0.106
	10	1.67	0.197	0.33	0.563	N/A^b^	N/A^b^	0.91	0.339	0.44	0.507	N/A^b^	N/A^b^
Triple negative (yes *vs* no)	All	**16.72**	**<0.0001**	**5.21**	**0.022**	3.80	0.051	**12.52**	**0.0004**	3.82	0.051	2.52	0.112
	1–9	**13.41**	**0.0003**	**4.92**	**0.027**	3.39	0.066	**10.29**	**0.001**	**4.44**	**0.035**	2.15	0.142
	10	2.48	0.115	0.08	0.774	N/A^b^^,^^c^	N/A^b^^,^^c^	1.32	0.250	0.03	0.854	N/A^b^^,^^c^	N/A^b^^,^^c^
Nodal status (+ *vs* −)	All	**19.14**	**<0.0001**	**5.33**	**0.021**	**9.55**	**0.002**	**15.92**	**<0.0001**	**11.90**	**0.0006**	2.07	0.150
	1–9	**16.96**	**<0.0001**	**4.72**	**0.030**	**9.21**	**0.002**	**12.42**	**0.0004**	**10.46**	**0.001**	1.87	0.171
	10	3.08	0.079	1.35	0.244	N/A^b^	N/A^b^	**4.62**	**0.032**	2.39	0.122	N/A^b^	N/A^b^

Abbreviations: ER=oestrogen receptor; WT=wild type.

Results are also detailed by deprivation groups 1–9 and 10.

a There were no *p53* mutation patients, who had Grade 1 tumours.

b All patients in deprivation group 10 without a *p53* mutation survived and remained disease free.

c All patients in deprivation group 10 without a *p53* mutation were free of triple-negative tumours. All analyses have one degree of freedom, and statistically significant results are highlighted in bold type.

**Table 3:** Results of Cox proportional hazards regression model, with death and recurrence as the response variables

	**Death**			**Recurrence**		
	***β***	***P***	**RR (95% CI)**	***β***	***P***	**RR (95% CI)**
Deprivation 10 (*vs* 1–9)	**1.81**	**0.0003**	**6.1** (**2.3–16.2)**	**1.31**	**0.003**	**3.7** (**1.6–8.9)**
Tumour size (large *vs* small)	**1.22**	**0.003**	**3.4** (**1.5–7.5)**	**0.83**	**0.012**	**2.3** (**1.2–4.4)**
ER (+ *vs* −)	**−2.50**	**0.041**	**0.1** (**<0.1–0.9)**	−1.28	0.056	0.3 (0.1–1.0)
*p53* mutation (*vs* WT)	0.67	0.114	1.9 (0.9–4.5)	**0.95**	**0.009**	**2.6** (**1.3–5.2)**
Tumour grade (3 *vs* 1–2)	0.90	0.087	2.5 (0.9–6.9)	0.18	0.677	1.2 (0.5–2.7)
Nodal status (+ *vs* −)	**1.32**	**0.005**	**3.7** (**1.5–9.5)**	**0.94**	**0.007**	**2.6** (**1.3–5.1)**
HER2 status (+ *vs* −)	−1.96	0.121	0.1 (<0.1–1.7)	−0.58	0.383	0.6 (0.2–2.1)
Triple negative (n *vs* y)	−1.54	0.229	0.2 (<0.1–2.6)	−0.74	0.325	0.5 (0.1–2.1)
						
Dep 10 & *p53* m (*v* rest)	**2.52**	**<0.0001**	**12.4** (**4.8–32.3)**	**2.18**	**<0.0001**	**8.8** (**3.6–21.7)**
Tumour size (large *vs* small)	**1.26**	**0.002**	**3.5** (**1.6–7.7)**	**0.90**	**0.005**	**2.5** (**1.3–4.7)**
ER (+ *vs* −)	**−2.65**	**0.021**	**0.1** (**<0.1–0.7)**	**−1.47**	**0.023**	**0.2** (**0.1–0.8)**
Tumour grade (3 *vs* 1–2)	**1.07**	**0.033**	**2.9** (**1.1–7.8)**	0.35	0.369	1.4 (0.7–3.0)
Nodal status (+ *vs* −)	**1.49**	**0.002**	**4.4** (**1.8–11.2)**	**1.05**	**0.002**	**2.9** (**1.5–5.6)**
HER2 status (+ *vs* −)	−1.57	0.184	0.2 (<0.1–2.1)	−0.23	0.718	0.8 (0.2–2.8)
Triple negative (n *vs* y)	−1.55	0.199	0.2 (<0.1–2.3)	−0.77	0.296	0.5 (0.1–2.0)

Abbreviations: ER=oestrogen receptor; RR=risk ratio; WT=wild type.

The upper models have deprivation and *p53* mutation status as individual binary input variables, whereas the lower models group deprivation category 10 patients that also have a *p53* mutation (*versus* all other patients) as a binary input variable. The G-statistic *χ*^2^⩾51.0 and *P*<0.0001 in each model, with the upper models having eight degrees of freedom and the lower models having seven. A strong predictor of the response variable (death or recurrence; left and right half of the table, respectively) is one that has a large *β*-coefficient, whether positive or negative, and a high degree of confidence in the coefficient, *P*⩽0.05. Such strong predictors are also backed by RR values with associated 95% confidence intervals that exclude unity. Indicators of strong predictors are highlighted in bold type.
